# Unanticipated Interfacial Redox Reaction in the NiO_x_ Transport Layer of Perovskite Light‐Emitting Diodes

**DOI:** 10.1002/advs.202510824

**Published:** 2025-11-27

**Authors:** Thi‐Hoai Do, Aswaghosh Loganathan, Yun‐Han Wu, Yaw‐Shyan Fu, Tzung‐Fang Guo

**Affiliations:** ^1^ Department of Photonics National Cheng Kung University Tainan 701 Taiwan ROC; ^2^ Department of Greenergy Technology National University of Tainan Tainan 700 Taiwan ROC; ^3^ Program on Key Materials Academy of Innovative Semiconductor and Sustainable Manufacturing National Cheng Kung University Tainan 701 Taiwan ROC; ^4^ Research Center for Applied Sciences (RCAS) Academia Sinica Taipei 115 Taiwan ROC; ^5^ Research Center for Critical Issues (RCCI) Academia Sinica Tainan 711 Taiwan ROC

**Keywords:** bias‐induced performance, metallic lead, NiO_x_/perovskite interface, oxidation‐reduction reaction

## Abstract

This study reveals an unanticipated interfacial redox reaction between the nickel oxide (NiO_x_) hole transport layer and perovskite active layer in organolead halide perovskite light‐emitting diodes (PeLEDs). Specifically, metallic nickel (Ni^0^) present in the NiO_x_ layer undergoes oxidation while lead ions (Pb^2+^) from the perovskite precursor are reduced, forming nickel (II) ions (Ni^2+^) and metallic lead (Pb^0^), as confirmed by X‐ray photoelectron spectroscopy (XPS). The formation of Pb^0^, a well‐known luminescence quenching center, is correlated with the significantly suppressed photoluminescence (PL) before biasing and the pronounced electroluminescence (EL) overshoot observed at the onset of electrical excitation. Introduction of an electrode interlayer, such as a polyvinyl carbazole (PVK), prevents direct contact between NiO_x_ and the perovskite, thereby effectively suppressing the detrimental redox reaction between them. This electrode interfacial modification eliminates Pb° formation, mitigates luminescence quenching, and suppresses EL overshoot. Moreover, the trap density at the perovskite interface is substantially reduced, and the deep trap distribution remains stable under bias. The findings offer critical insights into bias‐induced luminescence enhancement phenomena and present a reliable, scalable strategy to mitigate these phenomena, contributing to the development of advanced PeLEDs.

## Graphical abstract

This study presents the unanticipated interfacial redox reaction between NiO_x_ and perovskite, which is characterized by XPS. This redox reaction is one of the primary reasons for the bias‐induced instability of PeLEDs and is particularly suppressed by simply applying an interface layer between NiO_x_ and perovskite.

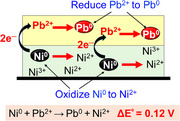

## 1 Introduction

Perovskite optoelectronics, including perovskite solar cells (PSCs) and light‐emitting diodes (PeLEDs), have progressed remarkably in terms of efficiency and stability over the past decade, driven by innovations in device design, materials chemistry, and interface engineering.^[^ ^1^, ^2^, ^3^ ^]^ Among various hole transport layers (HTLs), poly(3,4‐ethylenedioxythiophene):polystyrene sulfonate (PEDOT:PSS) has played a foundational role, with significant advances in conductivity, film morphology, and work function tuning over the years.^[^ ^4^, ^5^ ^]^ Despite these developments, the inherent acidity and hygroscopicity of PEDOT:PSS pose long‐term stability challenges for perovskite devices. In this context, p‐type nickel oxide (NiO_x_) has emerged as a robust alternative due to its wide bandgap, high carrier mobility, optical transparency, excellent thermal stability, tunable energy levels, and effective electron‐blocking characteristics.^[^ ^6^, ^7^, ^8^, ^9^, ^10^, ^11^ ^]^ Importantly, NiO_x_ offers a nonacidic, inorganic platform that can help address the degradation issues often associated with organic HTLs, making it especially attractive for enhancing the operational lifetime of PeLEDs and PSCs. Recent advancements have demonstrated that PeLEDs with NiO_x_ HTL can achieve a peak external quantum efficiency of up to 14.6%, along with significantly improved operational stability compared to devices utilizing conventional PEDOT:PSS HTLs.^[^ ^12^, ^13^ ^]^ Despite these advantages, direct interfacial interactions between NiO_x_ and the perovskite layer pose substantial challenges, adversely affecting device efficiency and long‐term stability.

Similar to its behavior in electrocatalytic and photoelectrocatalytic systems, NiO_x_ can engage in surface‐mediated redox reactions when in direct contact with perovskite layers. These interfacial reactions are governed by the chemical composition and electronic structure of NiO_x_. Previous studies have shown that Ni^3+^ species in NiO_x_ can react with Sn^2+^ from perovskite materials, or the highly oxidized Ni species (Ni^3+^ or higher) can interact with methylammonium iodide and formamidinium iodide, leading to interfacial degradation.^[^ ^14^, ^15^ ^]^ However, a unified understanding of these reaction mechanisms remains elusive, and the implications of such interfacial chemistry on energy conversion processes or optoelectronic performance are not yet fully understood. Various approaches, including surface treatment of NiO_x_,^[^ ^16^, ^17^, ^18^, ^19^ ^]^ elemental doping,^[^ ^20^, ^21^, ^22^ ^]^ and surface passivation strategies^[^ ^23^, ^24^, ^25^, ^26^ ^]^ have been explored to improve the physicochemical properties of NiO_x_, thereby enhancing the efficiency of PeLEDs and PSCs. Nevertheless, the mechanistic understanding and general principles guiding the selection of effective passivation or modification strategies for NiO_x_ remain areas of active research.

In our previous research using “bare” NiO_x_ HTL, we achieved high luminance exceeding 70 000 cd m^−2^.^[^ ^27^ ^]^ However, we observed a marked increase in electroluminescence (EL) intensity under constant bias application, associated with a non‐linear current density–luminance (*J*–*L*) relationship. Initially, we attributed these phenomena to ion migration within the methylammonium lead bromide (MAPbBr_3_) layer. To address this, we introduced choline chloride (Ch.Cl) as a grain boundary passivation agent.^[^ ^28^ ^]^ While this strategy successfully suppressed the EL overshoot and restored linearity in the *J–L* curve, the photoluminescence (PL) intensity of the Ch.Cl‐passivated perovskite film after biasing remained approximately twice as high as its initial value. This persistent PL enhancement suggests the involvement of an additional factor likely originating from interfacial processes at the NiO_x_/perovskite interface that contributes to the observed bias‐induced performance.

In this work, we employ X‐ray photoelectron spectroscopy (XPS) to investigate the interfacial reaction between NiO_x_ and the perovskite layer in PeLEDs. Our analysis reveals a spontaneous redox reaction between metallic nickel (Ni^0^) from NiO_x_ and lead ions (Pb^2+^), described by the equation: Ni^0^ + Pb^2+^ → Ni^2+^ + Pb^0^, with a standard electrode potential ΔE° = 0.12 V. This reaction leads to the formation of metallic lead (Pb^0^), which acts as a luminescence quenching center and likely accounts for both the suppressed PL intensity before bias application and the EL overshoot observed at the onset of biasing. To mitigate these effects, we introduce an electrode interlayer, such as polyvinyl carbazole (PVK), between NiO_x_ and perovskite or replace NiO_x_ altogether. This interfacial modification effectively prevents direct contact and suppresses the interfacial redox reaction between NiO_x_ and perovskite. In addition, we conduct capacitance analysis to probe and concretize the negative effect of the NiO_x_/perovskite interface and the benefit of the PVK interlayer on the interfacial charge dynamics under applied bias. Our model is further validated by introducing various organic interlayers and extending it to different perovskite systems, including quasi‐2D perovskites. These findings are significant as they confirm a fundamental origin of bias‐induced instabilities commonly observed in PeLEDs and offer a straightforward approach to effectively mitigate them.

## 2 Results and Discussion

**Figure 1:**
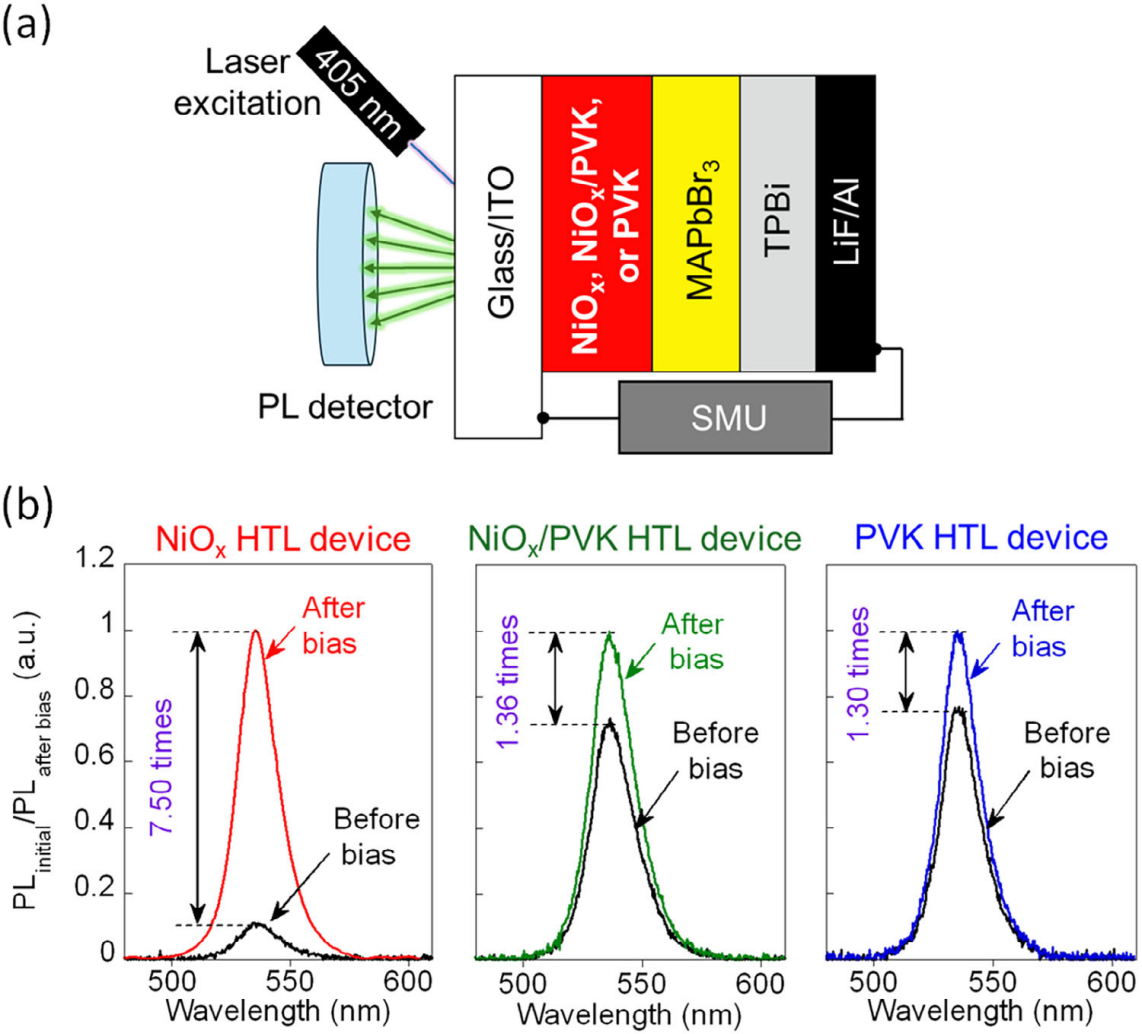
a) General scheme of PeLEDs in this study. b) Normalized PL intensity from MAPbBr_3_ film before and after bias (0–6 V in 0.1 V increments) of MAPbBr_3_ PeLEDs with NiO_x_ HTL (left), NiO_x_/PVK HTL (center), and PVK HTL (right).

To investigate the influence of the NiO_x_/perovskite interface on bias‐induced performance, we fabricated PeLEDs incorporating different HTL configurations, as shown in **Figure** **1**a. The standard device architecture features a control MAPbBr_3_ emissive layer positioned between a 1,3,5‐tris(1‐phenyl‐1*H*‐benzimidazol‐2‐yl)benzene (TPBi) electron transport layer and the following HTL configurations: i) NiO_x_ alone (NiO_x_ HTL), ii) NiO_x_ with a PVK interlayer (NiO_x_/PVK HTL), and iii) PVK alone, replacing NiO_x_ entirely (PVK HTL). All devices employ indium tin oxide (ITO) as the anode and lithium fluoride/aluminum (LiF/Al) as the cathode. To examine the modulation of PL properties in response to applied bias, PL spectra were recorded by irradiating a 405 nm excitation laser onto the emissive area (0.06 cm^2^) of MAPbBr_3_ PeLEDs from the glass/ITO side both before and after the application of voltage (0–6 V in 0.1 V increments).

Figure 1b presents the normalized PL spectra of the MAPbBr_3_ film before and after bias application on the device. Notably, the PL intensity of the device with the NiO_x_ HTL increases by a factor of 7.50 immediately after the biasing voltage is turned off.^[^ ^28^ ^]^ In contrast, this bias‐induced PL enhancement is obviously reduced in devices with interfacial modification: only 1.36‐fold and 1.30‐fold increases are observed in the NiO_x_/PVK HTL and PVK HTL devices, respectively. Furthermore, Figure S1 (Supporting Information) displays the scanning electron microscopy (SEM), UV–vis absorption, X‐ray diffraction (XRD), and XPS Pb4f analysis for the control MAPbBr_3_ films deposited on these different HTLs. These characterizations reveal no significant differences in MAPbBr_3_ in term of film morphology, crystallinity, or surface chemical composition across the samples.^[^ ^27^, ^28^, ^29^, ^30^ ^]^ Collectively, these results support our assumption that the NiO_x_/perovskite interface plays a critical role in governing the bias‐induced PL behavior. Additionally, the remaining PL enhancement in NiO_x_/PVK HTL and PVK/HTL devices is attributed to the ionic properties of the perovskite. Figure S2 (Supporting Information) shows that this will be further suppressed by directly applying a minimal necessary amount of passivation agent – Ch.Cl to the perovskite layer.

**Figure 2:**
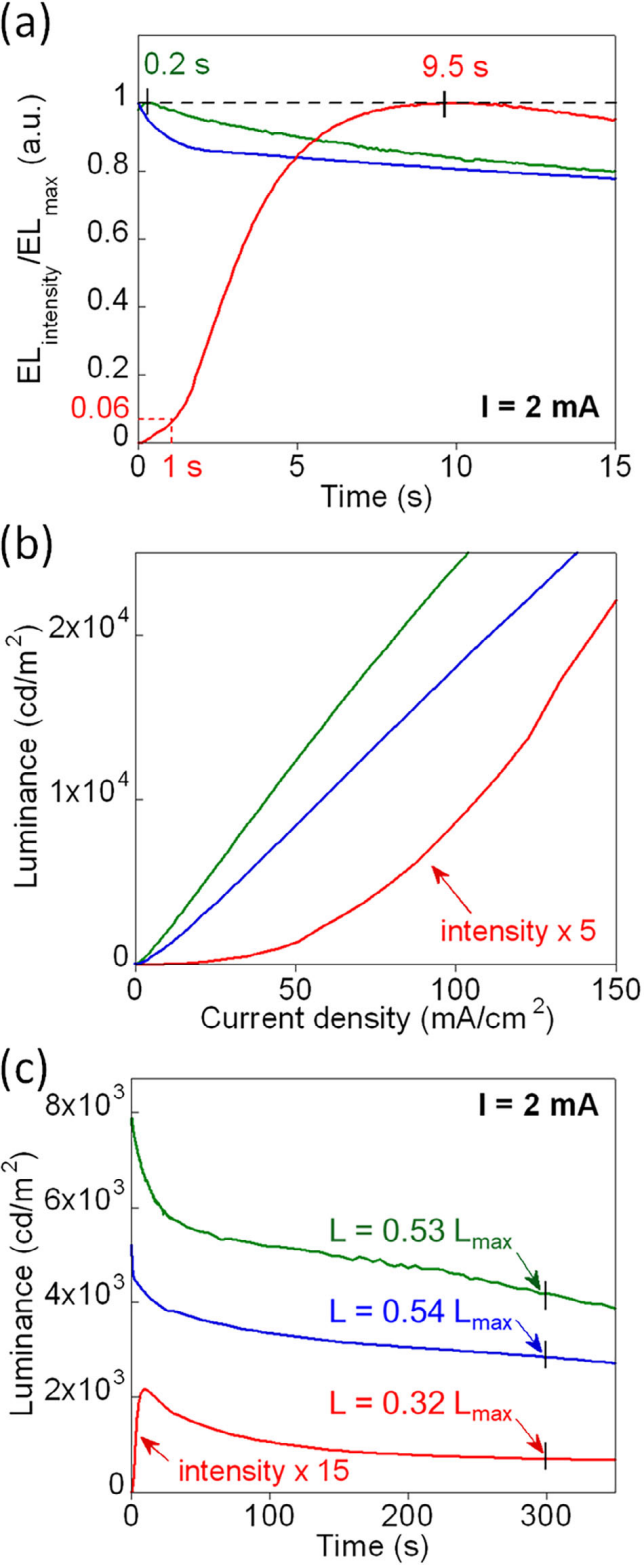
a) Normalized time‐dependent EL intensity; b) *J–L* characteristics, c) operational stability for MAPbBr_3_ PeLEDs with NiO_x_ HTL (red), NiO_x_/PVK HTL (green), and PVK HTL (blue). In (a) and (c), devices are operated at a constant current of 2 mA (≈33.3 mA cm^−2^).

In addition to modulating optical properties, electrical bias also affects the electronic performance of PeLEDs, particularly their EL behavior. A transient phenomenon characterized by an initial sharp increase and subsequent decrease or stabilization in EL intensity following the application of a constant electrical bias, commonly referred to as EL overshoot, is observed.^[^ ^31^, ^32^ ^]^ To investigate the correlation between this phenomenon and the NiO_x_/perovskite interface, we monitored the time‐dependent EL response under a constant driving current of 2 mA (≈33.3 mA cm^−2^), as shown in **Figure** **2**a. In the NiO_x_ HTL device, the EL intensity conspicuously rises in response to the applied bias. Within the first second, the EL reaches only ≈6% (or 0.06) of its peak value. The intensity continuously increases, eventually reaching its maximum at ≈9.5 s. In contrast, devices with NiO_x_/PVK and PVK HTLs achieve their peak EL intensities almost instantaneously within 0.2 s or less, indicating a significant reduction in the overshoot effect. This trend is consistent with the *J*–*L* characteristics shown in Figure 2b, where the NiO_x_ HTL device displays a non‐linear *J*–*L* relationship, deviating from the behavior typical of high‐quality LEDs.^[^ ^33^, ^34^, ^35^, ^36^ ^]^ In contrast, NiO_x_/PVK and PVK HTL devices exhibit a linear *J*–*L* dependence, indicating more stable and predictable charge recombination.

To assess the operational lifetime, Figure 2c presents the stress test result for NiO_x_ HTL, NiO_x_/PVK HTL, and PVK HTL devices under a constant current of 2 mA (≈33.3 mA cm^−2^). After 300 s of operation, the NiO_x_ HTL device retains only ≈32% of its maximum luminance (L = 0.32 L_max_, where L_max_ = 140 cd m^−2^). In contrast, the NiO_x_/PVK HTL and PVK HTL devices exhibit significantly higher luminance levels, with L_max_ = 7900 cd m^−2^ for NiO_x_/PVK and 5200 cd m^−2^ for PVK HTL devices. After the same duration, these devices maintained ≈53% and 54% of their initial luminance, corresponding to L = 0.53 and L = 0.54 L_max_, respectively. These findings clearly demonstrate that incorporating a PVK interlayer substantially enhances the EL intensity and operational stability of PeLEDs, possibly by mitigating detrimental interfacial reactions. Consistent with improved interfacial carrier dynamics, the suppression of bias‐induced PL enhancement and reduction of EL overshoot signify lower trap‐assisted recombination and diminished charge accumulation, which reduces electrical stress at the perovskite/HTL interface. This stabilization directly translates into higher luminance retention and longer device lifetime at a constant current. Therefore, the increase in device lifetime results is likely to align with the reduction of bias‐induced PL enhancement in Figure 1b and the suppression of EL overshoot in Figure 2a. Additional data presented in Figure S3 (Supporting Information), including *V–L* curves, current efficiency (CE), and EL emission profiles, further confirm the improved electronic performance of devices incorporating electrode interlayer PVK. These observations emphasize that the NiO_x_/perovskite interface also influences the electronic characteristics of the device, especially the intensity of EL emission. Introducing an electrode interlayer, such as PVK, effectively prevents direct contact between NiO_x_ and the perovskite layer, therefore mitigating the possible interfacial reactions and minimizing bias‐induced effects.

In our study, the NiO_x_ films were fabricated via a spin‐coating process of NiO_x_ precursor solution, followed by high‐temperature annealing in an oxygen‐rich environment, and subsequently exposed to ambient conditions during device fabrication and processing.^[^ ^37^ ^]^ Under these fabrication conditions, the reaction between NiO_x_ precursors is highly complex, leading to the formation of multiple nickel oxidation states. This chemical heterogeneity at the NiO_x_ surface can have significant implications for interfacial interactions with perovskite materials. To probe potential changes in the chemical and electronic structure of NiO_x_ upon contact with perovskite, we employed XPS, a powerful surface‐sensitive technique capable of resolving elemental states and interfacial reactions with high specificity.

**Figure 3:**
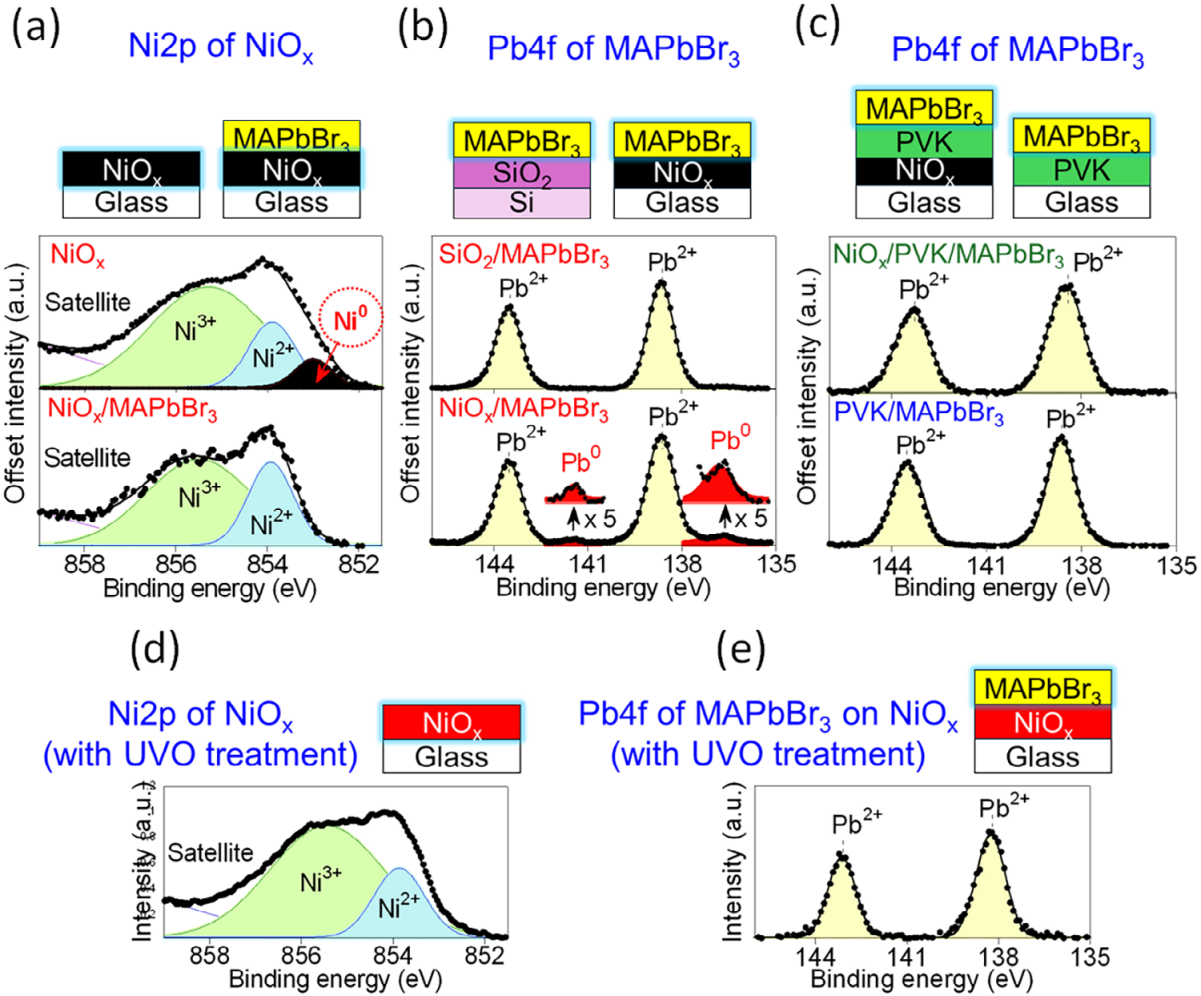
Schematic illustration of the measurement configuration and XPS analysis results: a) Ni2p spectra of NiO_x_ film before and after MAPbBr_3_ deposition; b) Pb4f spectra of MAPbBr_3_ deposited on SiO_2_ (300 nm SiO_2_ on the surface of n‐type silicon wafer), and as‐fabricated NiO_x_ HTL; c) Pb4f spectra of MAPbBr_3_ deposited on NiO_x_/PVK and PVK HTLs; d) Ni2p spectra of NiO_x_ film after UVO treatment for 30 min; e) Pb4f spectra of MAPbBr_3_ deposited on UVO‐treated NiO_x_ HTL. In these cases, the perovskite film thickness was below 10 nm.

**Figure** **3**a presents a schematic of the studied system and the XPS analysis of the Ni2p region for NiO_x_ films before and after perovskite deposition. The pristine NiO_x_ film exhibits characteristic peaks corresponding to Ni^3+^ and Ni^2+^ at binding energies of 855.4 and 853.9 eV, respectively, with a calculated Ni^3+^/Ni^2+^ ratio at 3.8:1.^[^ ^38^, ^39^, ^40^, ^41^, ^42^, ^43^ ^]^ Strikingly, a distinct Ni^0^ peak is observed at 852.9 eV. Despite the inherent complexity in the oxidation states of NiO_x_, the presence of Ni^0^ is well‐documented and has been reported in NiO_x_ films synthesized via spin‐coating, sputtering, and atomic layer deposition.^[^ ^44^, ^45^, ^46^, ^47^, ^48^ ^]^ However, this peak is usually neglected when introducing the NiO_x_ film. Notably, following the deposition of the thin layer of perovskite (thickness below 10 nm) onto the NiO_x_, the Ni^0^ peak disappears completely, leaving only Ni^3+^ and Ni^2+^ signals. Furthermore, the Ni^3+^/Ni^2+^ ratio decreases significantly to 2.4:1, indicating that Ni^0^ preferentially oxidizes to Ni^2+^ rather than Ni^3+^ upon interaction with the perovskite. These observations strongly suggest that Ni^0^ undergoes an interfacial redox reaction with the perovskite, leading to its oxidation and contributing to changes in the electronic structure of the NiO_x_ layer.

When the perovskite interacts with NiO_x_, changes in the perovskite composition are also observed. Figure 3b,c shows the XPS Pb4f spectra of the thin layer of perovskite films (thickness below 10 nm) deposited on different surfaces, namely SiO_2_ (300 nm SiO_2_ on the surface of n‐type silicon wafer), NiO_x_, NiO_x_/PVK, and PVK HTLs. Notably, in the spectrum of the perovskite film directly deposited on NiO_x_, new peaks emerge at binding energies of 141.3 and 136.7 eV, which are attributed to Pb^0^.^[^ ^49^, ^50^, ^51^, ^52^ ^]^ In contrast, these Pb^0^ peaks are all absent in films deposited on SiO_2_, NiO_x_/PVK, and PVK HTLs. These results provide strong evidence for the reduction of Pb^2+^ in the perovskite to Pb^0^ when in direct contact with NiO_x_. Moreover, the formation of Pb^0^ is also observed when PbI_2_ is thermally deposited on top of a nickel film, as shown in Figure S4 (Supporting Information). This observation further supports the occurrence of an interfacial redox reaction between Ni^0^ in NiO_x_ and Pb^2+^ from the perovskite, reinforcing the hypothesis of bidirectional chemical transformation at the NiO_x_/perovskite interface.

Based on the strong evidence from XPS, we propose that the chemical reaction at the NiO_x_/perovskite interface is a redox reaction involving Ni° from the NiO_x_ film and Pb^2+^ from the perovskite. To validate this hypothesis, we sought to eliminate Ni° from NiO_x_ film through prolonged UV‐ozone (UVO) treatment, which promotes surface oxidation. Figure 3d presents the Ni2p XPS spectrum result of NiO_x_ after UVO treatment, where the Ni^0^ peak is no longer detectable, and only Ni^3+^ and Ni^2+^ signals are observed. This confirms that we have successfully eliminated Ni^0^ by this treatment. Subsequently, a thin layer of perovskite film was deposited onto the UVO‐treated NiO_x_. As anticipated, XPS analysis of the Pb4f region, in Figure 3e, shows no evidence of Pb° formation in this perovskite film. The absence of Pb^0^ strongly supports our hypothesis that the interfacial redox reaction, specifically, the reduction of Pb^2+^ to Pb^0^, is directly driven by the presence of Ni^0^ in the NiO_x_ layer. Thus, eliminating Ni^0^ effectively prevents this undesirable reaction, reinforcing the proposed redox mechanism at the NiO_x_/perovskite interface.

**Figure 4:**
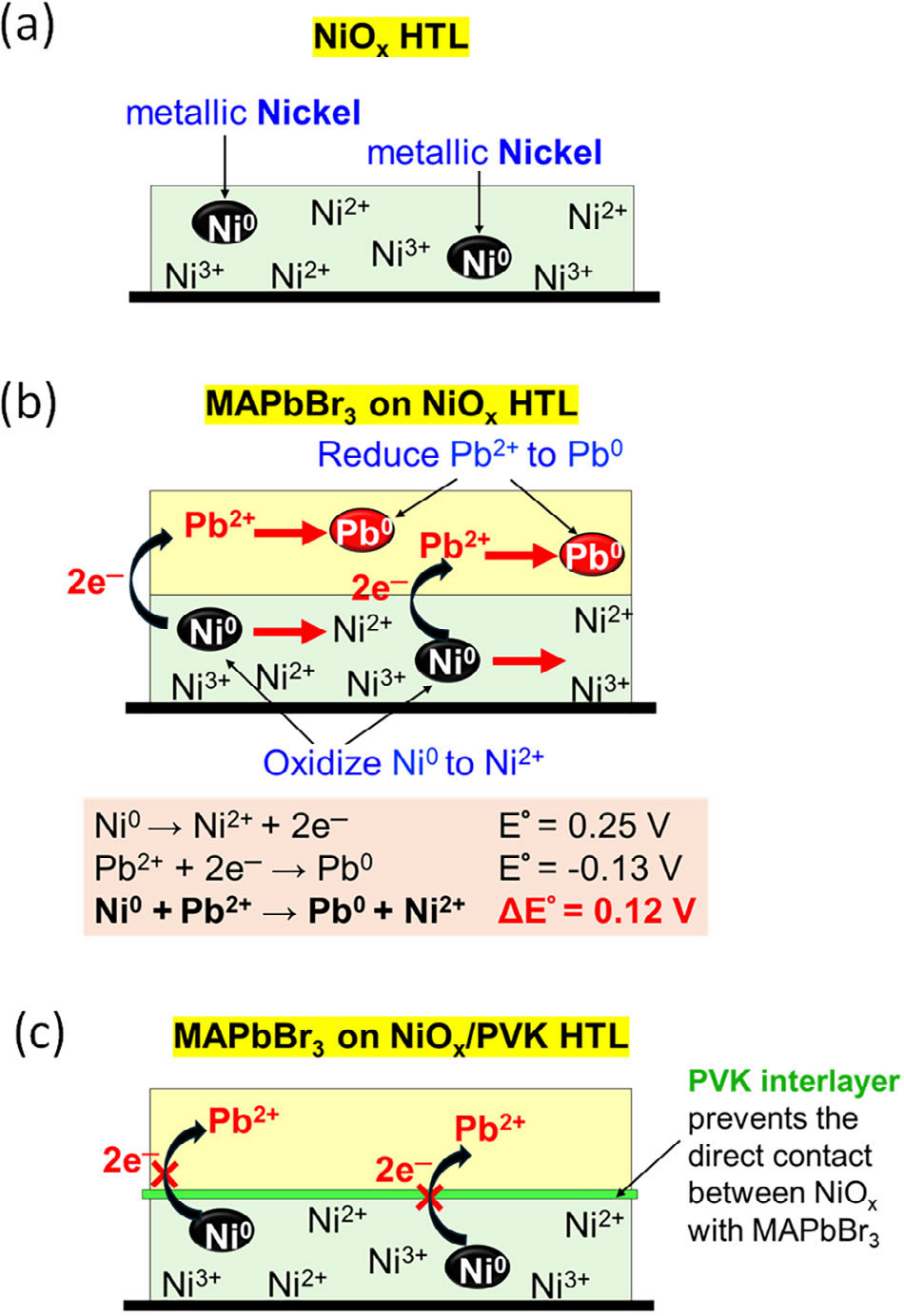
Schematic illustration of the proposed chemical interactions and the chemical states of key components: a) the as‐fabricated NiO_x_ HTL; b) the NiO_x_ HTL with MAPbBr_3_ film and associated interfacial redox reactions, where the equation represents the possible half‐reaction and the corresponding electron transfer between Ni^0^ and Pb^2+^; c) the NiO_x_/PVK HTL with MAPbBr_3_ film, where PVK is the interlayer.

Building upon the experimental evidence, we propose a mechanistic model illustrated in **Figure** **4**, which outlines the interfacial redox reaction and the corresponding chemical states of the involved components. Figure 4a depicts the as‐fabricated NiO_x_ film, which contains a mixture of Ni^3+^, Ni^2+^, and a small fraction of Ni^0^. Upon deposition of the perovskite layer, in Figure 4b, Pb^2+^ ion from the perovskite precursor solution comes into contact with Ni^0^ of NiO_x_, triggering a redox reaction as described by the equation: Ni^0^ + Pb^2+^ → Ni^2+^ + Pb^0^ (ΔE° = 0.12 V).^[^ ^53^ ^]^ Since ΔE° > 0, this redox reaction proceeds spontaneously. The resulting formation of Pb^0^ at the interface introduces non‐radiative recombination centers that act as luminescence quenching sites. This possibly explains the low PL observed prior to biasing and could be associated with the EL overshoot at the onset of bias application, as demonstrated in Figures 1b and 2a. Additionally, the presence of Pb^0^ potentially serves as the exciton trapping site and is related to the nonlinear *J*–*L* relationship observed in Figure 2b. In contrast, when a PVK interlayer is introduced between NiO_x_ and perovskite or when PVK fully replaces NiO_x_, direct contact between the perovskite and NiO_x_ is prevented, as shown in Figure 4c. As a result, the redox reaction between NiO_x_ and perovskite is effectively suppressed, and the chemical integrity of the perovskite layer adjacent to the PVK interface is preserved. The absence of interfacial Pb° formation likely allows the perovskite to maintain its intrinsic PL and EL performance, thereby reducing bias‐induced luminescence enhancement and restoring a linear *J*–*L* response, as shown in Figures 1b and 2b.

**Figure 5:**
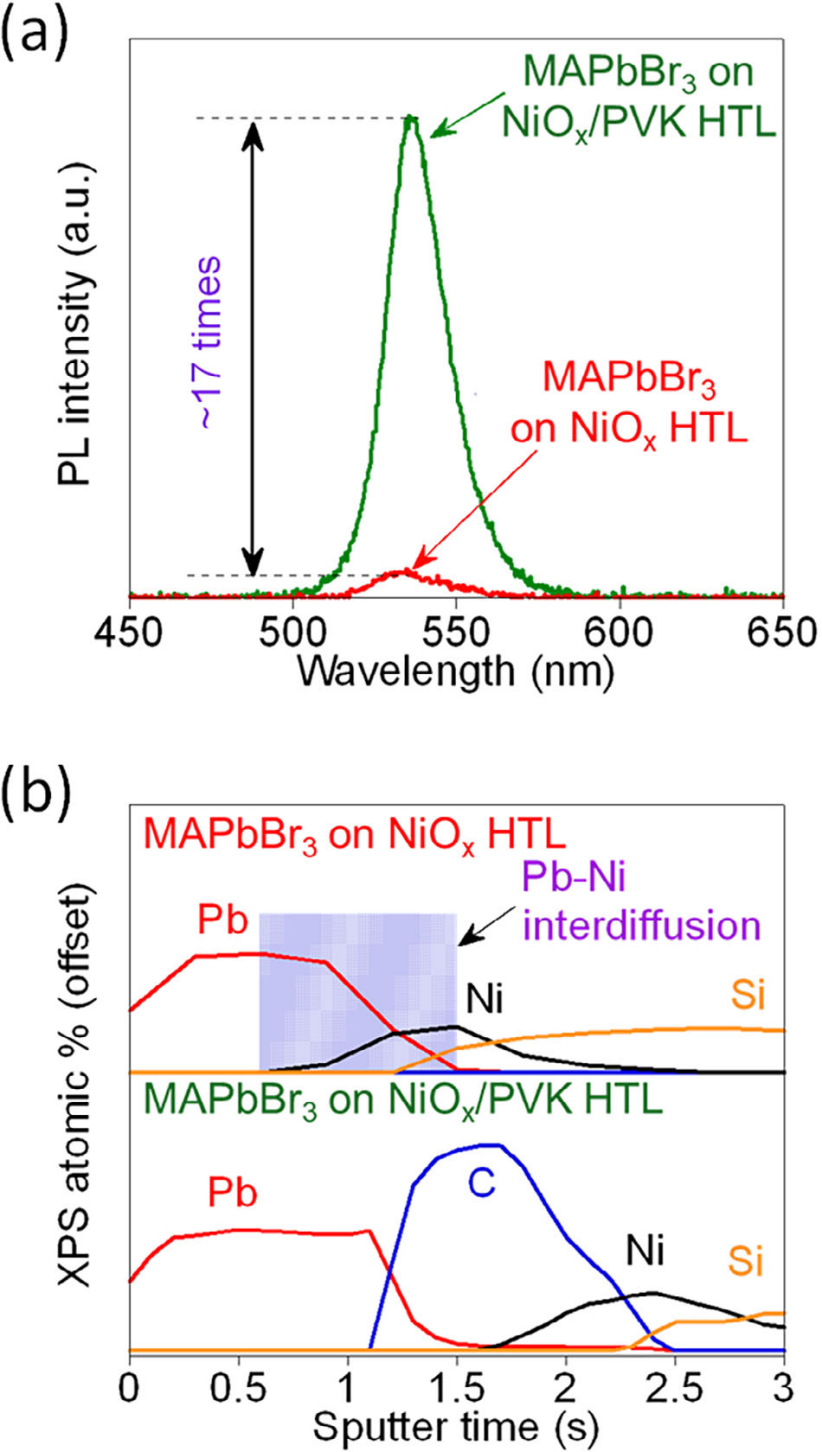
a) PL intensity of MAPbBr_3_ films deposited on NiO_x_ (red) and NiO_x_/PVK (green) HTLs. b) XPS depth profile of MAPbBr_3_ films on NiO_x_ (upper curves) and NiO_x_/PVK (lower curves) HTLs. In (b), the signals corresponding to Pb, C, Ni, and Si are attributed to the perovskite layer, PVK interlayer, NiO_x_ layer, and glass substrate, respectively.

To clarify whether the redox reaction occurring at the NiO_x_/perovskite interface could suppress the initial PL intensity and whether the introduction of a PVK interlayer could assist the perovskite film in preserving its intrinsic PL intensity, we investigated the initial PL intensities of perovskite films deposited on NiO_x_/PVK and NiO_x_ HTLs as illustrated in **Figure** **5**a. The PL intensity of the perovskite film on the NiO_x_/PVK HTL is ≈17 times higher compared to that on the NiO_x_ HTL. Furthermore, depth profile analysis using XPS was conducted to examine interfacial interactions, as shown in Figure 5b. The XPS results reveal substantial Ni–Pb interdiffusion at the interface between the perovskite and the NiO_x_ HTL. In contrast, this interdiffusion is effectively prevented when employing the PVK interlayer. This clearly demonstrates the capability of the PVK layer to inhibit detrimental interfacial reactions, which is in agreement with our proposed mechanism.

We additionally note that the PVK interlayer acts as a physical barrier, preventing direct contact between the NiO_x_ and perovskite layers. Supporting this observation, supplementary data Figure S5 (Supporting Information) reveals bias‐induced PL enhancement results of the MAPbBr_3_ PeLEDs with alternative electrode interlayers. In the results, when organic interlayers such as poly(4‐butylphenyldiphenylamine) (poly‐TPD), Ch.Cl, and (2‐(3,6‐dimethoxy‐9*H*‐carbazol‐9‐yl)ethyl) phosphonic acid (MeO‐2PACz), a self‐assembled molecule commonly used in PSCs, are applied, there is a significant reduction in bias‐induced PL enhancement. This indicates that PVK may be substituted with other materials capable of similarly blocking direct contact of NiO_x_ and perovskite, thereby preventing interface reaction between them, possibly eliminating the formation of Pb^0^.

**Figure 6:**
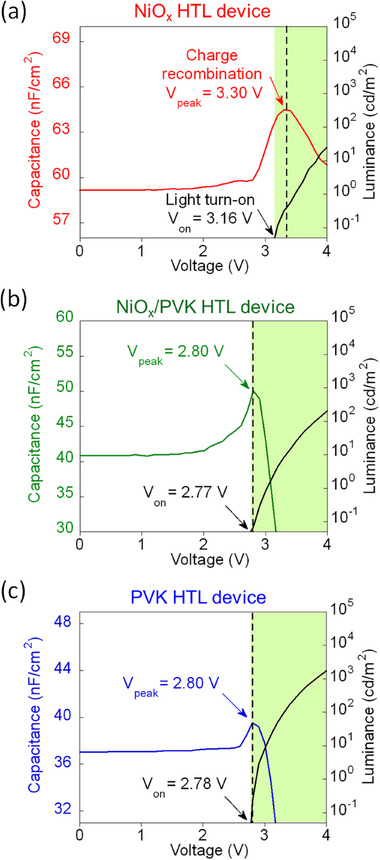
The *C–V–L* curve (f = 1 kHz, AC = 30 mV) of a MAPbBr_3_ PeLEDs with a) NiO_x_ HTL, b) NiO_x_/PVK HTL, and c) PVK HTL.

Avoiding the formation of Pb^0^ is crucial for improving the electrical processes of devices.^[^ ^54^, ^55^, ^56^, ^57^ ^]^ To further investigate the interfacial dynamics under an applied electric field, we conducted capacitance–voltage–luminance (*C*–*V*–*L*) measurements, as presented in **Figure** **6**. The voltage at which the capacitance begins to sharply decrease, indicating the onset of optimal radiative recombination, is termed as V_peak_. In our measurements, V_peak_ was observed at 3.30 V for the device using the NiO_x_ HTL, as shown in Figure 6a. In contrast, V_peak_ was notably lower at 2.80 V for devices incorporating either the NiO_x_/PVK HTL (Figure 6b) or solely the PVK HTL (Figure 6c). A lower V_peak_ corresponds to optimal charge recombination occurring at lower applied voltages, signifying enhanced charge injection efficiency and faster recombination rates when the PVK interlayer is introduced.

Additionally, the voltage at which devices begin emitting detectable light, termed V_on_ was recorded at ≈3.16 V for the NiO_x_ HTL device. However, V_on_ was significantly reduced to ≈2.77 and 2.78 V for NiO_x_/PVK HTL and PVK HTL devices. A lower V_on_ implies superior charge injection capabilities and reduced energy losses. Ideally, the V_on_ should coincide closely with V_peak_.^[^ ^58^ ^]^ Notably, for the NiO_x_ HTL device, V_on_ (3.16 V) occurs distinctly earlier than V_peak_ (3.30 V), while for both NiO_x_/PVK HTL and PVK HTL devices, V_on_ closely matches V_peak_ (V_peak_ is 2.80 V, V_on_ is 2.77 and 2.78 V for NiO_x_/PVK HTL and PVK HTL devices, respectively). This suggests that a substantial charge remains accumulated at the NiO_x_/perovskite interface and highlights the difference in the carrier dynamics when the PVK interlayer is introduced.^[^ ^59^, ^60^ ^]^ Figure S6 (Supporting Information) shows the energy‐level alignment across different HTL configurations,^[^ ^61^, ^62^ ^]^ providing insight into the observed electrical behavior. In the NiO_x_ HTL device, the hole injection barrier between the NiO_x_ and the MAPbBr_3_ perovskite is ≈0.6 eV, which hampers efficient hole injection and leads to charge accumulation at the interface. In contrast, PVK exhibits a well‐aligned HOMO level, resulting in a nearly negligible injection barrier to the perovskite layer. When PVK is inserted between NiO_x_ and MAPbBr_3_, it forms a cascade structure that reduces the effective injection barrier. This alignment facilitates more efficient hole transport and minimizes interfacial charge buildup. Consequently, the reduced V_peak_ observed in devices containing PVK either as a standalone HTL or as an interlayer can be attributed to improved energy‐level alignment and stabilized hole injection, despite PVK's relatively lower mobility. This improved interfacial alignment leads to synchronized charge injection and recombination, resulting in minimal separation between V_on_ and V_peak_ in NiO_x_/PVK and PVK‐only devices. Furthermore, the reduced charge accumulation and balanced carrier dynamics at the interface in PVK‐containing devices can stabilize the injection process. As a result, devices with NiO_x_/PVK HTLs exhibit superior CE and improved operational characteristics compared to PVK‐only and NiO_x_‐only devices.

**Figure 7:**
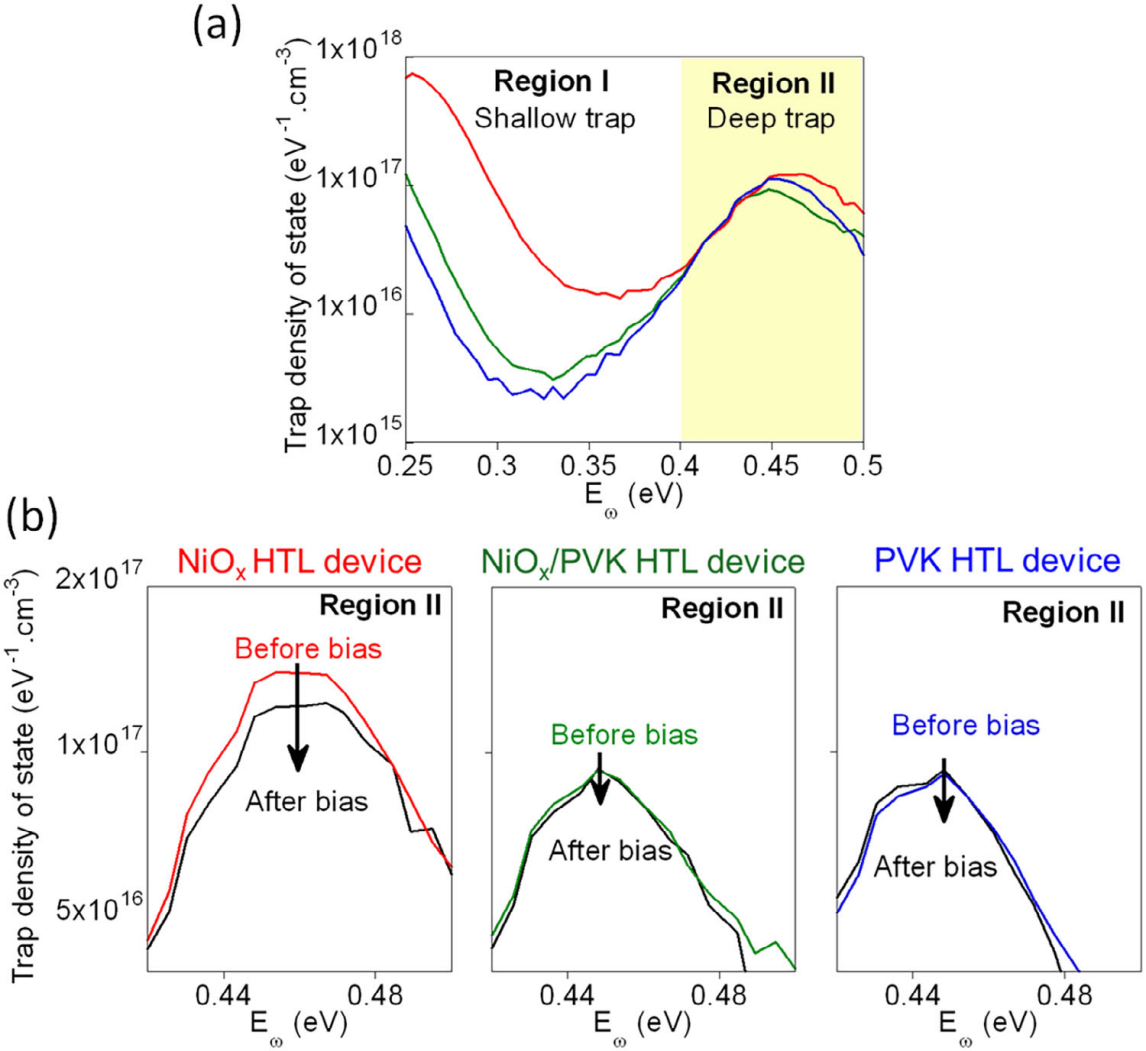
a) tDOS as a function of E_ω_ (0.25–0.5 eV) of the MAPbBr_3_ PeLEDs with NiO_x_ HTL (red), NiO_x_/PVK HTL (green), and PVK HTL (blue). b) Deep trap distribution before and after application of an external bias (0–6 V in 0.1 V increments) of MAPbBr_3_ PeLEDs with NiO_x_ HTL (left), NiO_x_/PVK HTL (center), and PVK HTL (right).

Charge accumulation at the NiO_x_/perovskite interface after device operation may arise from several factors, including the presence of surface trap states on NiO_x_, deep traps induced by Pb^0^, and charges involved in additional electrochemical reactions, etc. To investigate this further, we analyzed the trap density of states (tDOS) as a function of energy (E_ω_) for devices employing NiO_x_ HTL, NiO_x_/PVK HTL, and PVK HTL, as shown in **Figure** **7**a. The tDOS analysis reveals two distinct trap‐state regions: Region I (below 0.4 eV), corresponding to shallow traps, and Region II (above 0.4 eV), associated with deep traps in the perovskite films. The NiO_x_ HTL device exhibits shallow trap densities approximately one order of magnitude higher compared to devices with NiO_x_/PVK HTL and PVK HTL. This increase is primarily attributed to numerous dangling bonds on the NiO_x_ surface and alterations in perovskite surface defects directly contacting NiO_x_.^[^ ^31^ ^]^ Since the bulk perovskite layers remain unaffected by different HTLs, deep‐trap levels in Region II are expected to be similar across devices. However, our results show that the NiO_x_ HTL device also exhibits higher deep‐trap densities compared to the NiO_x_/PVK HTL and PVK HTL devices. This elevated density likely results from Pb° formation at the NiO_x_/perovskite interface.^[^ ^54^, ^55^, ^56^, ^57^ ^]^

We further examined how bias voltage affects deep‐trap states, as illustrated in Figure 7b. The devices were pre‐biased 0–6 V in 0.1 V increments. Notably, the NiO_x_ HTL device demonstrates a significant decrease in deep‐trap density following bias application, whereas no substantial changes occur in either NiO_x_/PVK HTL or PVK HTL devices. These observations suggest that the bias‐induced reduction in deep traps may be linked to the suppression or re‐oxidation of Pb^0^ in the NiO_x_ HTL device under forward bias. Supporting evidence comes from the consistent trap distribution before and after bias application in super yellow phenyl‐substituted poly(*p*‐phenylene vinylene), SY‐PPV LEDs, Figure S7 (Supporting Information), which have inherently low trap densities and lack Pb^0^. In summary, bias‐induced reduction in deep‐trap density exclusively occurs in the NiO_x_ HTL device, indicating that Pb^0^ or Pb^0^‐related deep traps are likely re‐oxidized or de‐trapped during device operation. This hypothesis explains the enhanced EL and PL intensities observed after biasing, as previously described in Figures 1b and 2a. Although direct evidence for Pb^0^ re‐oxidation remains to be demonstrated, the presented data strongly support this proposed mechanism. Figure S8 (Supporting Information) illustrates the energetic location of the Pb^0^‐related deep trap states within the band structure of MAPbBr_3_, based on reported literature.^[^ ^63^, ^64^, ^65^ ^]^ The Pb^0^‐related defects are believed to reside near the mid‐gap, significantly above the valence band edge (−5.9 eV), and act as a deep hole trap in the un‐biased state and possibly undergo de‐trap or re‐oxidation upon biasing. In contrast, shallow traps are located closer to the valence band maximum.^[^ ^66^, ^67^, ^68^ ^]^

**Figure 8:**
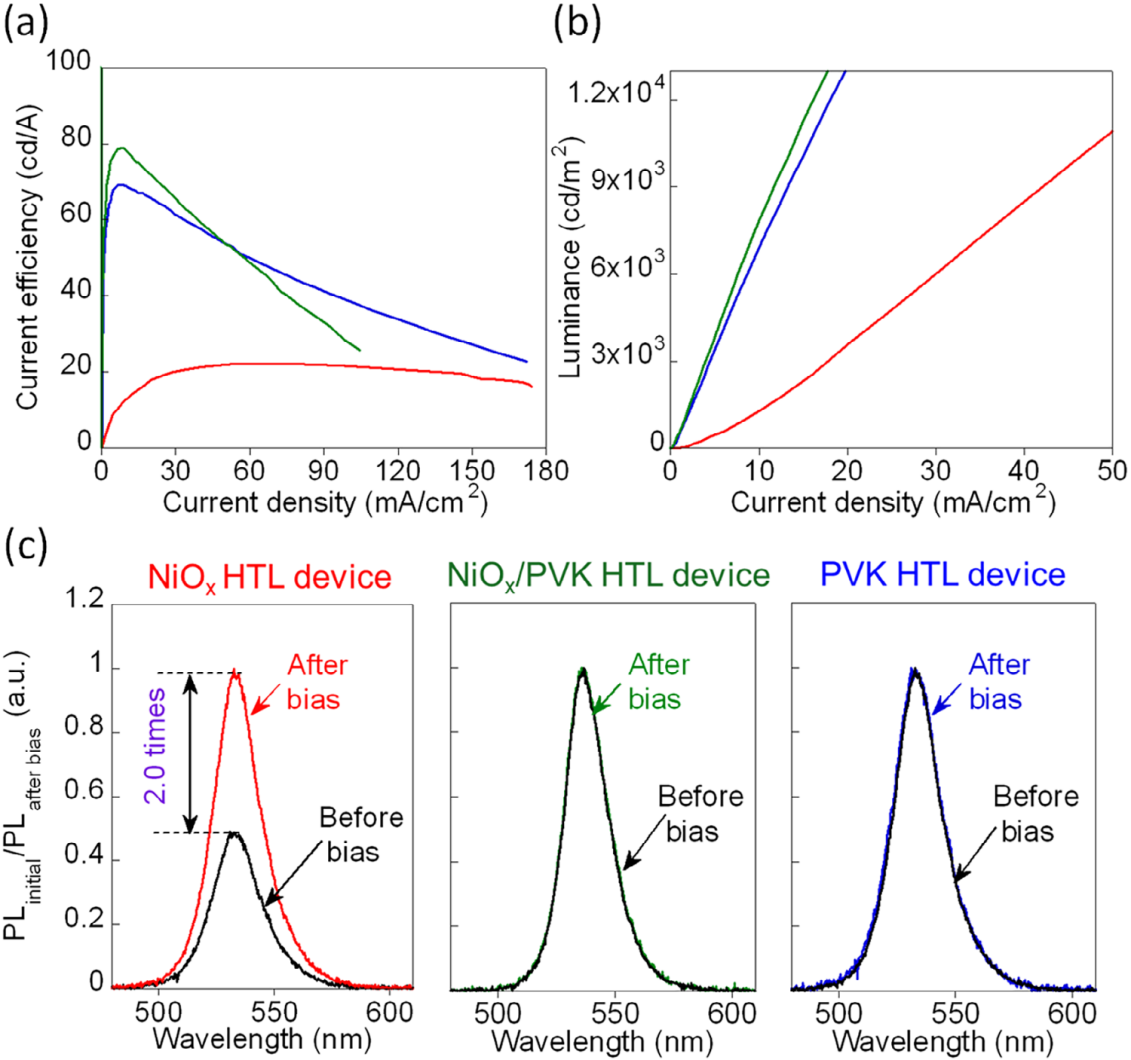
a) CE versus current density; b) the *J–L* characteristic of the quasi‐2D PEA_2_(FAPbBr_3_)_2_PbBr_4_ PeLEDs with NiO_x_ HTL (red), NiO_x_/PVK HTL (green), and PVK HTL (blue). c) Normalized PL intensity before and after bias (0–6 V in 0.1 V increments) of quasi‐2D PeLEDs with NiO_x_ HTL (left), NiO_x_/PVK HTL (center), and PVK HTL (right).

To further validate our proposed mechanism, we extended our investigation from the 3D MAPbBr_3_ system to the quasi‐2D PEA_2_(FAPbBr_3_)_2_PbBr_4_ perovskite. Similar to the 3D devices, quasi‐2D PeLEDs were fabricated using NiO_x_ HTL, NiO_x_/PVK HTL, and PVK HTL configurations. Consistent with our previous findings, the quasi‐2D devices also showed significantly enhanced CE when the interfacial redox reaction was suppressed by the introduction of the PVK interlayer, as illustrated in **Figure** **8**a. Specifically, the maximum CE improved remarkably from 22 cd A^−1^ for the NiO_x_ HTL device to 79 and 70 cd A^−1^ for the NiO_x_/PVK HTL and PVK HTL devices, respectively. This high enhancement in CE arises from the inherently higher photoluminescence quantum yield and the favorable intrinsic properties of quasi‐2D perovskites compared to their 3D counterparts.

Moreover, as presented in Figure 8b, the NiO_x_ HTL quasi‐2D device exhibited nonlinear *J–L* behavior, whereas devices incorporating either the NiO_x_/PVK HTL or the PVK HTL exhibited a linear relationship. Although the *J–L* characteristic remains nonlinear for the NiO_x_ HTL quasi‐2D device, its slope is closer to linear behavior compared to the NiO_x_ HTL device with 3D perovskite, as observed in Figure 2b. Furthermore, Figure 8c illustrates that the NiO_x_ HTL quasi‐2D device experienced a 2.0‐fold increase in PL intensity after bias application, whereas devices incorporating PVK interlayer showed no notable changes. Although PL enhancement is still observed following bias application in the NiO_x_ HTL quasi‐2D device, the magnitude of this enhancement is significantly reduced compared to the NiO_x_ HTL device using the 3D perovskite, which exhibits a 7.5‐fold increase in PL intensity (Figure 1b). The more linear *J–L* slope and reduced PL enhancement after bias can be attributed to the higher ion‐migration barrier in quasi‐2D perovskites compared to 3D perovskites, as well as the natural barrier provided by organic ligands present in quasi‐2D structures, partially mitigating direct contact between NiO_x_ and perovskite.^[^ ^69^, ^70^ ^]^ Collectively, these results confirm that the redox reaction occurring at the NiO_x_/perovskite interface presents significant challenges across various perovskite systems, extending beyond just 3D MAPbBr_3_. The incorporation of low‐dimensional perovskites combined with an effective interlayer like PVK successfully mitigates bias‐induced instabilities, representing a promising strategy toward achieving stable and highly efficient PeLEDs upon further optimization.

Kim et al.^[^ ^71^ ^]^ previously reported that ion migration contributes to EL overshoot in 3D PeLEDs and suggested that reducing ion migration by transitioning from 3D to quasi‐2D perovskite structures is an effective approach. Our previous findings also support that ion migration indeed plays a role in bias‐induced enhancement phenomena such as EL overshoot.^[^ ^28^ ^]^ Nevertheless, despite the transition to quasi‐2D structures, an EL overshoot of 7.4% was still observed in Kim's study.^[^ ^71^ ^]^ In our previous work,^[^ ^28^ ^]^ a higher PL intensity still persisted after bias application, even after applying ion migration passivation. This indicates that ion migration is not the sole factor responsible for this phenomenon. Here, we explicitly demonstrate that the interface between NiO_x_ and perovskite substantially contributes to the bias‐induced enhancement. Additionally, we propose and validate a straightforward yet effective solution to eliminate these interfacial issues.

NiO_x_ holds significant promise as an HTL precisely because it is an inorganic material, offering the intrinsic potential for superior robustness and operational stability—a critical advantage for commercial viability. However, realizing this potential requires careful optimization. The electronic and interfacial properties of NiO_x_ are highly dependent on its stoichiometry; unoptimized, it leads to detrimental charge recombination and redox reactions with the perovskite. Therefore, the path forward involves a dual strategy: precise control of NiO_x_ stoichiometry paired with a charge‐regulating interlayer to decouple it from the perovskite. Such tailored interface engineering is paramount to minimizing power loss and finally unleashing the stable, high‐efficiency performance that inorganic HTLs like NiO_x_ can provide.

## 3 Conclusion

In summary, using XPS and capacitance analysis, we provided direct evidence for the redox reaction occurring between NiO_x_ and the perovskite layer. We linked this reaction to irregular PeLED behaviors, including nonlinear *J–L* characteristics, bias‐induced PL enhancement, and the overshoot of EL under constant bias conditions. By directly targeting the root cause of these instabilities associated with the NiO_x_ transport layer, our study offers practical guidelines for selecting appropriate interlayers to overcome these challenges. Finally, by combining quasi‐2D PEA_2_(FAPbBr_3_)_2_PbBr_4_ perovskites with a suitable interlayer, we successfully suppressed the bias‐induced phenomena, such as bias‐induced PL enhancement and EL overshoot under the electric bias. This approach represents a transformative strategy for the development of next‐generation high‐performance PeLEDs and related optoelectronic devices.

## Conflict of Interest

The authors declare no conflict of interest.

## Supporting information



Supporting Information

## Data Availability

The data that support the findings of this study are available from the corresponding author upon reasonable request.
