# Supplementary material for: Unanticipated Interfacial Redox Reaction in the NiOx Transport Layer of Perovskite Light‐Emitting Diodes
Source: Adv Sci (Weinh). 2025 Nov 27;13(9):e10824. doi: 10.1002/advs.202510824 (PMC12904025; doi:10.1002/advs.202510824)
Supplement: Supplementary file 1 — Supporting Information [file ADVS-13-e10824-s001.docx]

Supporting Information

**Unanticipated Interfacial Redox Reaction in the NiO_x_ Transport Layer of Perovskite Light-Emitting Diodes**

*Thi-Hoai Do, Aswaghosh Loganathan, Yun-Han Wu, Yaw-Shyan Fu, Tzung-Fang Guo**

Thi-Hoai Do, Aswaghosh Loganathan, Yun-Han Wu, Tzung-Fang Guo

Department of Photonics, National Cheng Kung University, Tainan 701, Taiwan ROC

Yaw-Shyan Fu

Department of Greenergy Technology, National University of Tainan, Tainan 700, Taiwan ROC

Tzung-Fang Guo

Program on Key Materials, Academy of Innovative Semiconductor and Sustainable Manufacturing, National Cheng Kung University, Tainan 701, Taiwan ROC

Research Center for Applied Sciences (RCAS), Academia Sinica, Taipei 115, Taiwan ROC

Research Center for Critical Issues (RCCI), Academia Sinica, Tainan 711, Taiwan ROC

*Email: guotf@mail.ncku.edu.tw, guotf@as.edu.tw

Keywords: metallic lead, NiO_x_/perovskite interface, bias-induced performance, oxidation-reduction reaction.

**Experimental section**

***Materials and precursor preparation:***

*Material*

Methylammonium bromide (MABr), formamidinium bromide (FABr), phenethylammonium bromide (PEABr), and methylammonium chloride (MACl) were purchased from Greatcell Solar Materials (Australia). 1,3,5-tris(1-phenyl-1*H*-benzimidazol-2-yl)benzene (TPBi, >99.8%) was obtained from Lumtec (Taiwan). Chloroform (CHCl_3_, 99.0-99.4%) was purchased from Honeywell (USA). Polyvinyl carbazole (PVK, M_w_ ~1,100,000), lead (II) bromide (PbBr_2_, trace metals basis), choline chloride (Ch.Cl, ≥99%), dimethyl sulfoxide (DMSO, ≥99.7%), ethyl alcohol (ethanol, ≥99.45%), isopropyl alcohol (IPA, >99.5%), and chlorobenzene (CB, 99.8%) were purchased from Sigma-Aldrich (USA). Poly(4-butylphenyldiphenylamine) (poly-TPD) was purchased from Ossila (UK). A self-assembled molecule (2-(3,6-dimethoxy-9*H*-carbazol-9-yl)ethyl) phosphonic acid (MeO-2PACz, >98%) was purchased from TCI (Japan). All chemicals were used without further purification.

*Preparation of MAPbBr_3_ and passivated-MAPbBr_3_ precursor*

Control methylammonium lead bromide (MAPbBr_3_) precursor solution (1.23 M) was prepared by dissolving 0.15 g of MABr and 0.48 g of PbBr_2_ (1.04:1 molar ratio) in 1.07 mL of DMSO. The solution was stirred for 12 hours in a nitrogen-filled glove box, maintaining oxygen and moisture levels below 1.0 ppm. For thin-film fabrication (<10 nm), the control MAPbBr_3_ solution was diluted tenfold in DMSO to yield a 0.123 M concentration. The passivated-MAPbBr_3_ precursor solution was prepared by directly adding 0.58 wt% Ch.Cl (related to mass of PbBr_2_) into 1.07 ml control MAPbBr_3_ precursor solution and stirred overnight prior to use.

*Preparation of quasi-2D PEA_2_(FAPbBr_3_)_2_PbBr_4_ precursor*

Quasi-2D PEA_2_(FAPbBr_3_)_2_PbBr_4_ perovskite precursor was prepared by mixing 0.22 g of PbBr_2_, 0.05 g of FABr, 0.08 g of PEABr, and 0.004 g of MACl in 1 mL of DMSO. The mixture was stirred for 12 hours in a nitrogen-filled glove box prior to use.

*Preparation of NiO_x_ precursor*

Nickel oxide (NiO_x_) precursor solution was prepared by dissolving 0.46 g of nickel formate dihydrate (Alfa Aesar) in a mixture of 5 mL ethylene glycol (Sigma-Aldrich), 0.33 mL ethylenediamine (Alfa Aesar), and 0.30 mL ethanolamine (J.T. Baker). The solution was stirred at room temperature for 24 hours and filtered through a 0.45 µm nylon filter before use.

***Device Fabrication:***

*Fabrication of MAPbBr_3_ and passivated-MAPbBr_3_ PeLEDs*

Glass/ITO substrates were cleaned and treated with UV-ozone (Model 42, Jelight, USA) for 25 minutes to increase surface hydrophilicity. The NiO_x_ precursor was spin-coated at 4500 rpm for 90 seconds, followed by annealing in a tubular furnace at 400 °C for 10 minutes under an oxygen atmosphere (10 liters per minute) to form NiO_x_ film. This substrate was then transferred into a nitrogen-filled glove box (MBRAUN) with moisture and oxygen level below 1.0 ppm for further fabrication. Following, MAPbBr_3_ film (or passivated-MAPbBr_3_) was deposited by spin-coating 100 µL of precursor solution in a two-step process: 500 rpm for 7 seconds, then 4000 rpm for 70 seconds. At 43 seconds in the second step, 250 µL of CHCl_3_ was dropped as an anti-solvent. The film was annealed at 70 °C for 10 minutes. Subsequently, TPBi (350 Å, 0.3–0.5 Å/s), LiF (10 Å, 0.1 Å/s), and Al (800 Å, 2 Å/s) were thermally evaporated under ultrahigh vacuum (5 × 10⁻⁶ Torr). The active area of the device was 0.06 cm^2^.

*Fabrication of quasi-2D PeLEDs*

For the fabrication of quasi-2D PeLEDs, 100 µL of the PEA_2_(FAPbBr_3_)_2_PbBr_4_ precursor solution was spin-coated on glass/ITO/NiO_x_ substrates at 3000 rpm for 120 seconds. At 60 seconds, 100 µL of CB was dropped as the anti-solvent. The film was annealed at 90 °C for 60 minutes. TPBi, LiF, and Al layers were then deposited using similar processes to MAPbBr_3_ PeLEDs.

*Fabrication of PVK layer/interlayer*

For NiO_x_/PVK and PVK HTLs, 60 µL of PVK (4 mg/mL in CB) was spin-coated onto glass/ITO/NiO_x_ or glass/ITO substrates at 1000 rpm for 60 seconds, followed by annealing at 120 °C for 20 minutes. The thicknesses of the NiO_x_, NiO_x_/PVK, and PVK HTLs were estimated to be approximately 10 ± 3 nm, 25 ± 5 nm, and 12 ± 3 nm, respectively, using a Tencor Alpha-Step 500 surface profiler.

*Fabrication of poly-TPD interlayer*

For the poly-TPD electrode interlayer, 60 µL poly-TPD solution (4 mg/mL in CB) was spin-coated onto the glass/ITO/NiO_x_ substrate at 1000 rpm for 60 seconds, followed by annealing at 120 °C for 20 minutes.

*Fabrication of MeO-2PACz interlayer*

The MeO-2PACz electrode interlayer was fabricated by spin-coating 100 µL of a MeO-2PACz solution (0.1 mM in ethanol) on a glass/ITO/NiO_x_ substrate at 3000 rpm for 30 seconds, and the substrate surface was rinsed three times with 100 µL of ethanol, followed by annealing at 100 °C for 10 minutes.

*Fabrication of Ch.Cl interlayer*

The Ch.Cl electrode interlayer was fabricated by spin-coating 60 µL of a Ch.Cl solution (0.02 g Ch.Cl in 1 ml IPA) on a glass/ITO/NiO_x_ substrate at 4000 rpm for 30 seconds.

*UVO treatment NiO_x_ film*

For UVO-treated NiO_x_, the fabricated NiO_x_ film was placed in the UV-ozone chamber for 30 minutes to convert Ni^0^ into a higher oxidation state.

*Fabrication of SY-PPV LEDs*

For SY-PPV LEDs, a hole transport layer of poly(3,4-ethylenedioxythiophene):(polystyrene sulfonate) (PEDOT:PSS, AI4083, Baytron P, Bayer AG) was filtered through a 0.45 µm PVDF filter and spin-coated onto the patterned glass/ITO substrates at 4000 rpm. The films were annealed at 150 °C for 30 minutes. Super yellow phenyl-substituted poly(*p*-phenylene vinylene) (SY-PPV, PDY-132, Merck, 0.5% in toluene) was spin-coated at 5000 rpm for 60 seconds inside a nitrogen-filled glove box. Then, thermal evaporation deposited LiF (10 Å) and Al (800 Å).

**Characterization**

Current density*–*voltage*–*luminance (*J–V–L*) characteristic and electroluminescence (EL) overshoot measurements were conducted using a Keithley 2400 source meter and a Keithley Pico multimeter. EL intensity was recorded by a silicon photodiode (Hamamatsu S2387, Japan) and calibrated using a PR655 spectrophotometer (Photo Research, USA). Steady-state photoluminescence (PL) spectra were recorded using a custom-built setup with an Ocean Optics USB2000 miniature spectrometer and a 405 nm laser source (LSR405NL-80, Lasever Inc., China). For bias-dependent PL, devices were additionally pre-biased at 0–6 V in 0.1 V increments by Keysight 4145B Semiconductor Parameter Analyzer. A shadow mask (0.06 cm^2^) was used to confine the PL measurement area. Capacitance measurements were performed using an HP Agilent 4284A LCR meter over a frequency range of 20 Hz to 1 MHz with a 30 mV AC bias. For bias-dependent trap density of states (tDOS) measurements, the devices were DC pre-biased from 0 to 6 V in 0.1 V increments with an integration time of 100 µs. For capacitance–voltage–luminance (*C–V–L*) measurements, the devices were biased from 0 to 6 V in 0.1 V steps at a fixed frequency of 1 kHz, using the same integration time of 100 µs. All electrical and optical measurements were conducted in a nitrogen-filled glove box with oxygen and moisture levels below 1.0 ppm. X-ray diffraction (XRD) patterns were collected using a Bruker D8 Advance diffractometer (Bragg-Brentano geometry) with Cu Kα radiation (25 mA, 40 kV), scanning from 10° to 45° (2θ) at a rate of 0.10° s⁻^1^. The scanning electron microscopes (SEM) images were obtained using a Supra 55 Scanning Electron Microscope (Zeiss) (GEMINI Technology, UK). UV-visible absorption spectra were obtained using a Hitachi U4100 spectrometer. X-ray photoelectron spectroscopy (XPS) measurements were performed using a PHI VersaProbe 4 instrument with a monochromatic Al Kα source (20.6 W, 100 µm spot size, 45° incidence angle, 0.03 eV resolution). Importantly, the mobile vacuum chamber was applied to transport the sample without exposure to the ambient. Depth profiling was carried out using argon ion sputtering, and samples were not rotated during etching.

**Figure S1:** (a) The SEM top view (upper image) and cross-section view (bottom image) of the control MAPbBr_3_ film on NiO_x_ HTL (left), NiO_x_/PVK HTL (center), and PVK HTL (right). (b) The UV absorption, and (c) the XRD pattern of control MAPbBr_3_ film on NiO_x_ HTL (red), NiO_x_/PVK HTL (green), and PVK HTL (blue). (d) The XPS Pb4f of control MAPbBr_3_ film on NiO_x_ HTL (top), NiO_x_/PVK HTL (center), and PVK HTL (bottom).


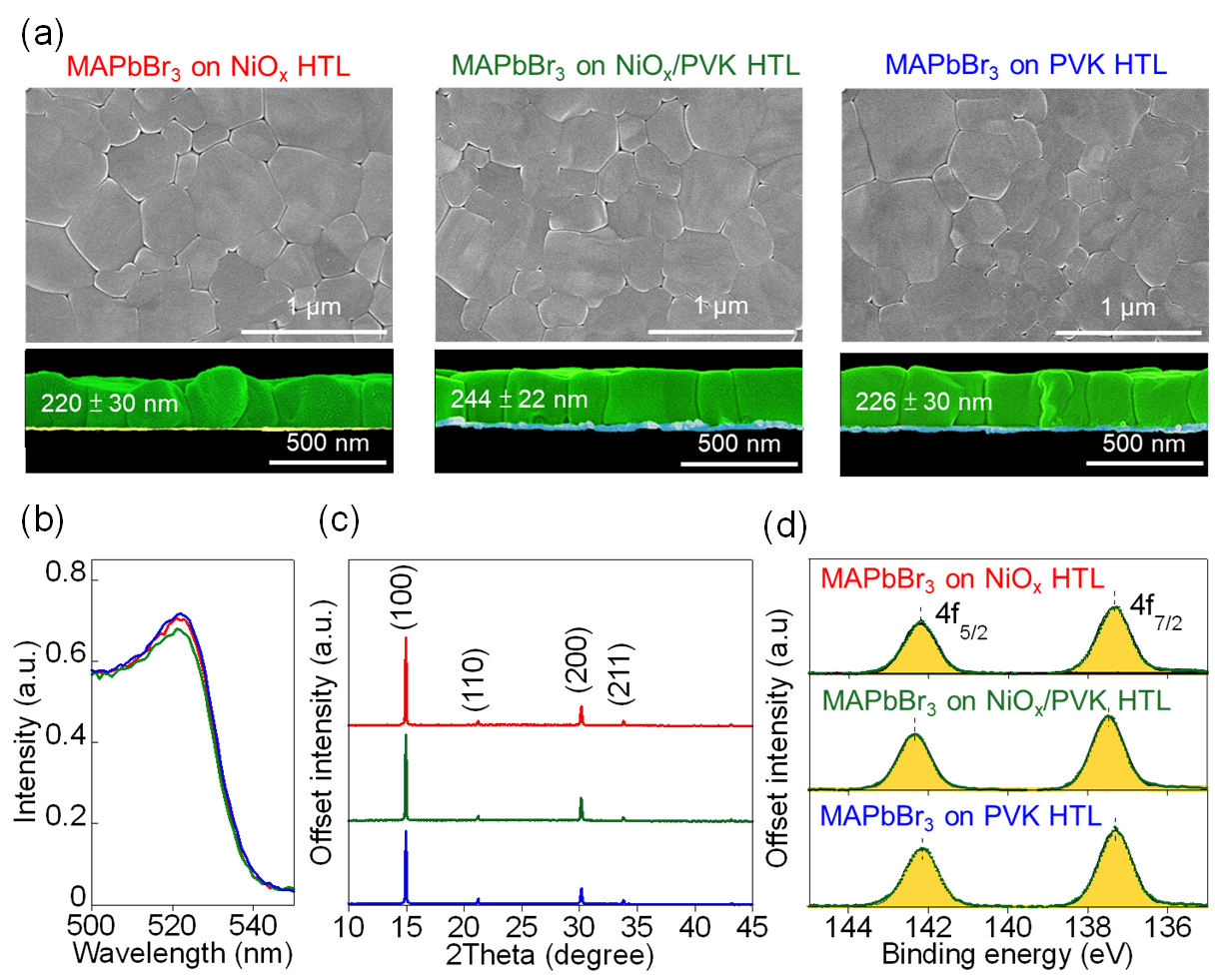


This analysis confirms that the performance variations in PeLEDs originate from interfacial electronic effects, not from differences in the bulk perovskite properties. As shown in **Figure S1**, the perovskite films deposited on different HTLs exhibit nearly identical morphology (grain size, thickness), crystallinity (XRD), composition (XPS of Pb4f), and UV absorption. Since these bulk and structural characteristics are unchanged, the significant differences in the device’s output characteristic must be attributed to the specific charge injection dynamics and possible chemical interactions at the NiO_x_/perovskite interface.**Figure S2:** (a) The normalized PL intensity from 0.58 wt% Ch.Cl passivated-MAPbBr_3_ film before and after bias (0–6 V in 0.1 V increments) of passivated-MAPbBr_3_ PeLEDs with device configuration of ITO/NiO_x_/PVK/passivated-MAPbBr_3_/TPBi/LiF/Al (left), and ITO/PVK/passivated-MAPbBr_3_/TPBi/LiF/Al (right), and (b) the *J–L–V* curves of passivated-MAPbBr_3_ PeLEDs with NiO_x_/PVK HTL (green), and PVK HTL (blue).


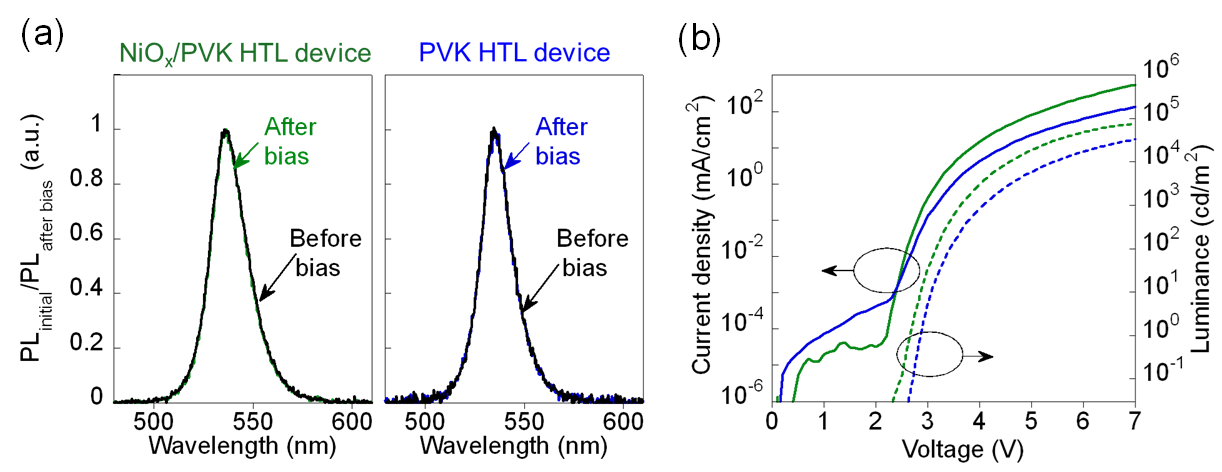


**Figure S2(a)** shows that the bias-induced PL has been significantly suppressed with the passivation of Ch.Cl. We emphasize that the amount of Ch.Cl used here (0.58 wt%), which is significantly lower than in our previous study (3 wt%) when the NiO_x_/perovskite interface is not considered as a factor influencing bias-induced enhancement.^[S1]^ This supports the idea that bias-induced PL is related to both factors: the interface between NiO_x_/perovskite and the intrinsic ionic effects in the perovskite layer. The device’s electrical output characteristic is also improved after perovskite is passivated. **Figure S2(b)** shows the *J–L–V* characteristics of the passivated-MAPbBr_3_ device with different NiO_x_/PVK and PVK HTLs, reducing the V_on_ to 2.30 V and 2.64 V, while also improving the current density and maximum luminance.**Figure S3:** (a) The *V–L* curves, (b) CE versus current density curves, and (c) normalized EL intensity of the MAPbBr_3_ PeLEDs with NiO_x_ HTL (red), NiO_x_/PVK HTL (green), and PVK HTL (blue).


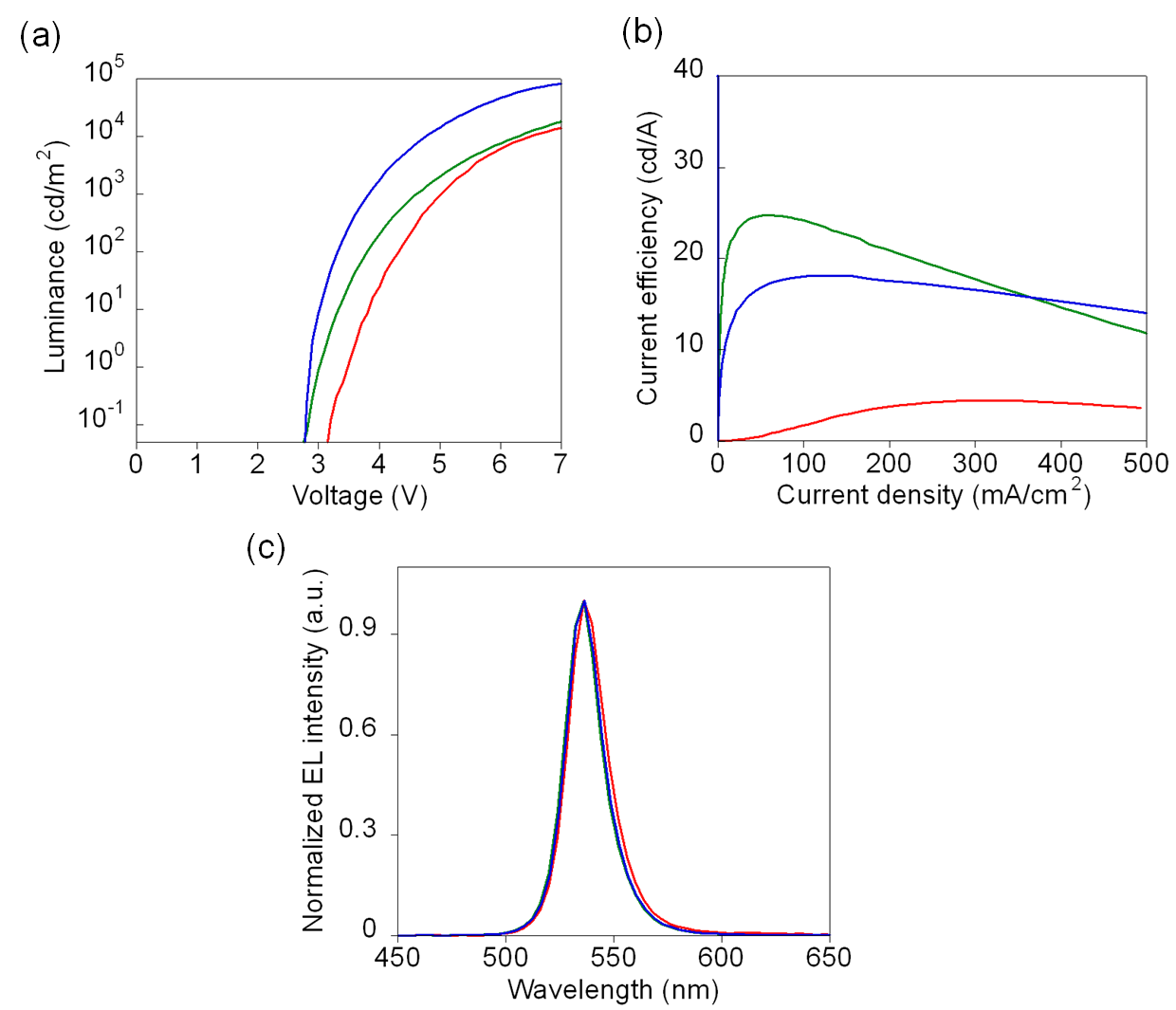


The significantly lower electrical performance of the NiO_x_ HTL device suggests that direct NiO_x_/perovskite contact leads to substantial interfacial losses. This is evidenced in **Figure S3(a)** by its higher turn-on voltage (3.16 V) and lower luminance (13,991 cd/m^2^) compared to NiO_x_/PVK and PVK devices (2.8 V, >18,000 cd/m^2^). The corresponding CE in **Figure S3(b)** confirms this trend, with the NiO_x_ device peaking at only 4.4 cd/A, significantly lower than the 24.8 and 18.1 cd/A of the other architectures. The EL spectra in **Figure S3(c)** remain consistent across devices, reconfirming that the perovskite’s bulk film does not significantly change by varying different HTLs.

**Figure S4:** The XPS Pb4f spectra in thermally evaporated PbI_2_ (thickness ~ 10 nm) on different substrates of nickel (red) and on quartz (blue).

As shown in **Figure S4**, the formation of metallic Pb^0^ is observed when a 10 nm PbI_2_ layer is fabricated on a nickel film (red), but it is not observed when PbI_2_ is deposited on quartz (blue). This demonstrates that the reduction of Pb^2+^ to Pb^0^ is a spontaneous reaction at the Ni interface, even in the solid state.

**Figure S5:** (a) Summarized bias-induced PL enhancement of device configuration glass/ITO/NiO_x_/electrode interlayer/MAPbBr_3_/TPBi/LiF/Al. The electrode interlayer is varied from no interlayer (NiO_x_, blue) to an electrode buffer layer of PVK (NiO_x_/PVK, light blue), poly-TPD (NiO_x_/Poly-TPD, green), choline chloride (NiO_x_/Ch.Cl, orange), and MeO-2PACz (NiO_x_/MeO-2PACz, brown). (b) Chemical structure of those interlayers.


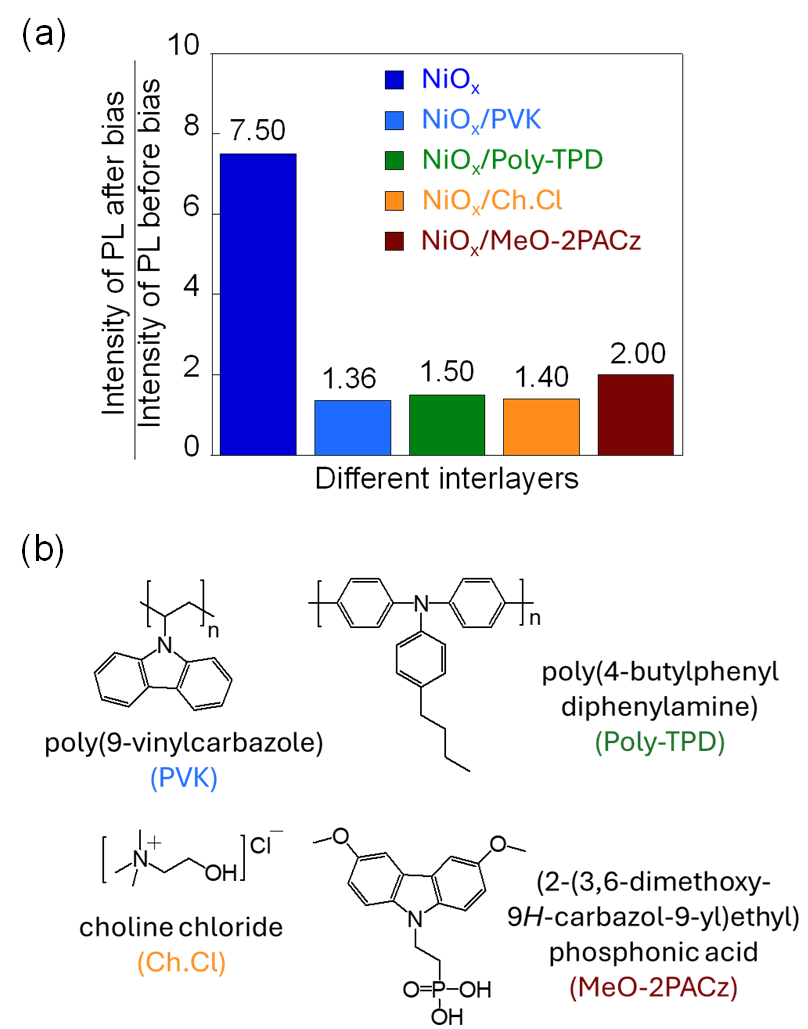


**Figure S5** demonstrates that the suppression of bias-induced PL enhancement is a general benefit of inserting any effective interlayer, not a unique property of PVK. While the NiO_x_-only device shows a 7.50-fold PL increase, all devices with an interlayer—NiO_x_/PVK (1.36-fold), NiO_x_/poly-TPD (1.50-fold), NiO_x_/Ch.Cl (1.40-fold), and NiO_x_/MeO-2PACz (2.00-fold)—exhibit a drastically reduced effect. This confirms that the primary role of the interlayer is to physically and electronically decouple the perovskite from the NiO_x_ surface, and that a variety of materials capable of this function can serve as viable alternatives to PVK.

**Figure S6:** Energy-level alignment diagram of individual layers used in the PeLEDs with (a) NiO_x_ HTL, (b) PVK HTL, and (c) NiO_x_/PVK HTL based on reported UPS and optical data from representative literature sources.


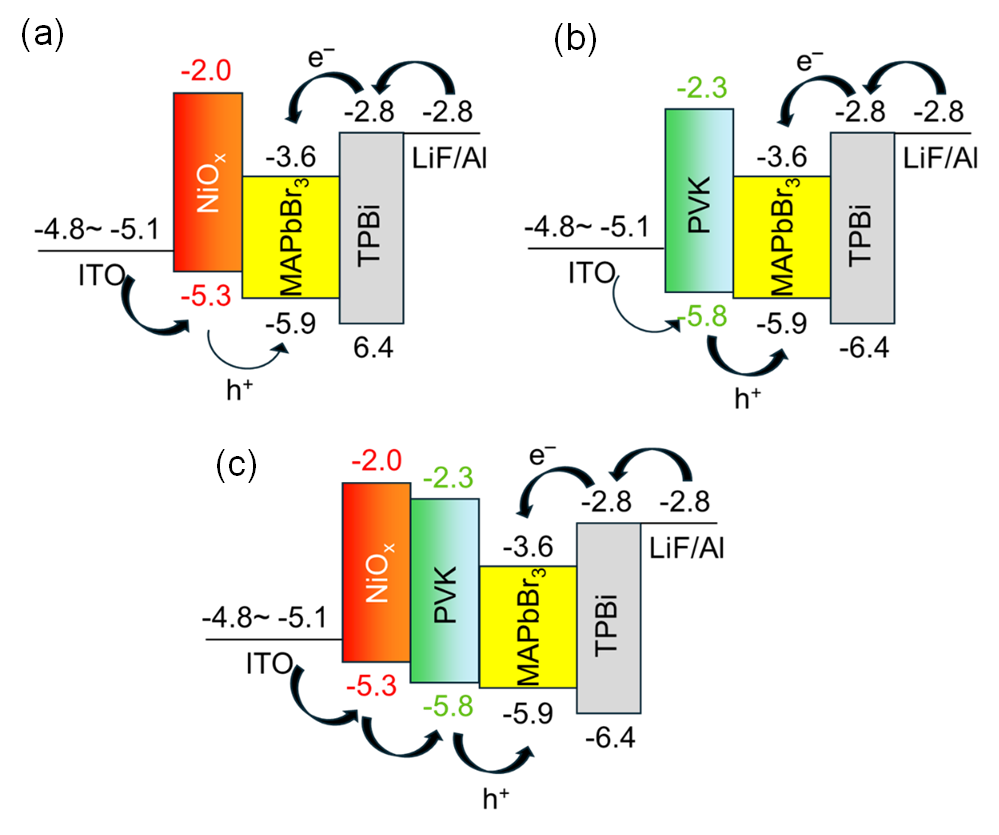


**Figure S6** illustrates the energy-level alignment across various HTL configurations,^[S2, S3]^ providing insight into the observed electrical behavior. In the NiO_x_ HTL device, the hole injection barrier between the NiO_x_ (HOMO: –5.3 eV) and the MAPbBr_3_ perovskite (valence band: –5.9 eV) is approximately –0.6 eV, which hampers efficient hole injection and leads to charge accumulation at the interface. In contrast, PVK exhibits a well-aligned HOMO level (–5.8 eV), resulting in a remarkably negligible injection barrier to the perovskite layer. When PVK is inserted between NiO_x_ and MAPbBr_3_, it forms a cascade structure that reduces the effective injection barrier. This alignment facilitates more efficient hole transport and minimizes interfacial charge buildup. Consequently, the reduced V_peak_ observed in devices containing PVK either as a standalone HTL or as an interlayer can be attributed to improved energy-level alignment and stabilized hole injection, despite PVK's relatively lower mobility.

**Figure S7: (**a) The tDOS distribution as a function of E_ω_ of SY-PPV LED with device structure: glass/ITO/PEDOT:PSS/SY-PPV/LiF/Al. (b) Deep trap distribution of SY-PPV LED before (continued line) and after bias (dashed line) of 0–6 V in 0.1 V increments.

**
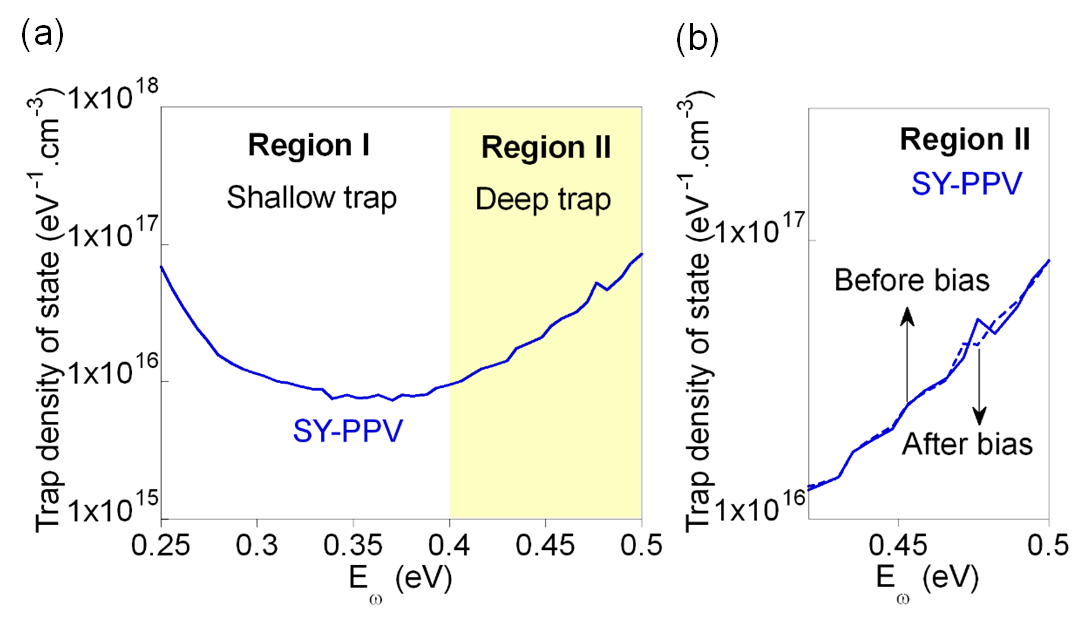
**

**Figure S7(a)** illustrates the tDOS distribution for the SY-PPV LED. Both the shallow and deep traps of SY-PPV range from 10^-16^–10^-17^ eV^-1^.cm^-3^ reflects the inherently low trap density of SY-PPV material. **Figure S7(b)** displays the deep-trap distribution before and after bias, presenting no apparent change in the deep-trap distribution after bias.

**Figure S8:** Schematic representation of the electronic density of states in MAPbBr_3_, illustrating the Pb^0^-related deep trap located near the mid-gap region based on representative reports.


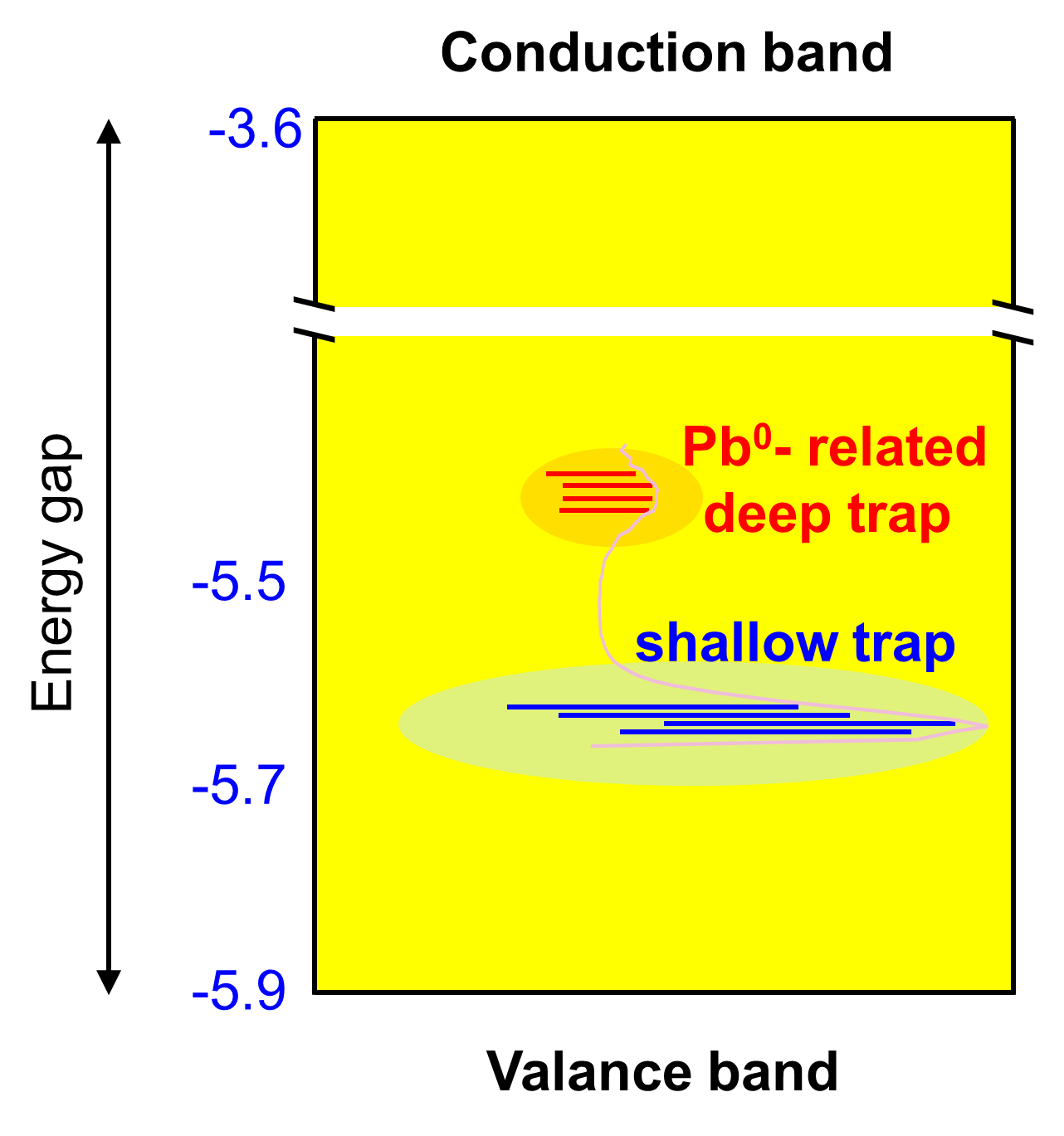


This illustration highlights the likely location of the deep trap state associated with Pb^0^-related deep traps near the mid-gap,^[S4-S6]^ between the valence band (–5.9 eV) and conduction band (–3.6 eV) of MAPbBr_3_. This defect state can function as a hole trap in the unbiased state and possibly de-trapping or undergo re-oxidation upon biasing.

**Reference**

[S1] T. L. Shen, A. Loganathan, T. H. Do, C.-M. Wu, Y.-T. Chen, Z.-J. Chen, N.-C. Chiu, C.-H. Shih, H.-C. Wang, J.-H. Chou, Y.-Y. Hsu, C.-C. Liu, Y.-C. Chang, Y.-S. Fu, W.-C. Lai, P. Chen, T.-C. Wen, T.-F. Guo, *Adv. Opt. Mater.* **2022**, *10*, 2101439.

[S2] Y.-K. Chih, J.-C. Wang, R.-T. Yang, C.-C. Liu, Y.-C. Chang, Y.-S. Fu, W.-C. Lai, P. Chen, T.-C. Wen, Y.-C. Huang, C.-S. Tsao, T.-F. Guo, *Adv. Mater.* **2016**, *28*, 8687.

[S3] X. Xiao, K. Wang, T. Ye, R. Cai, Z. Ren, D. Wu, X. Qu, J. Sun, S. Ding, X. W. Sun, W. C. H. Choy, *Commun. Mater.* **2020**, *1*, 81.

[S4] M.-Y. Hao, H.-Y. Wang, Y. Wang, Y. Qin, J.-P. Zhang, X.-C. Ai, *J. Power Sources* **2020**, *479*, 229077.

[S5] K. Kearney, G. Seo, T. Matsushima, C. Adachi, E. Ertekin, A. Rockett, *J. Am. Chem. Soc.* **2018**, *140*, 15735.

[S6] H. Jin, E. Debroye, M. Keshavarz, I. G. Scheblykin, M. B. J. Roeffaers, J. Hofkens, J. A. Steele, *Mater. Horiz.* **2020**, *7*, 397.
